# Rethinking ‘Rational Imitation’ in 14-Month-Old Infants: A Perceptual Distraction Approach

**DOI:** 10.1371/journal.pone.0032563

**Published:** 2012-03-14

**Authors:** Miriam Beisert, Norbert Zmyj, Roman Liepelt, Franziska Jung, Wolfgang Prinz, Moritz M. Daum

**Affiliations:** 1 Max-Planck-Institute for Human Cognitive and Brain Sciences, Leipzig, Germany; 2 Ruhr-Universität Bochum, Bochum, Germany; 3 Westfälische Wilhelms University, Münster, Germany; 4 Maastricht University, Maastricht, The Netherlands; Università di Parma, Italy

## Abstract

In their widely noticed study, Gergely, Bekkering, and Király (2002) showed that 14-month-old infants imitated an unusual action only if the model freely chose to perform this action and not if the choice of the action could be ascribed to external constraints. They attributed this kind of selective imitation to the infants' capacity of understanding the principle of rational action. In the current paper, we present evidence that a simpler approach of perceptual distraction may be more appropriate to explain their results. When we manipulated the saliency of context stimuli in the two original conditions, the results were exactly opposite to what rational imitation predicts. Based on these findings, we reject the claim that the notion of rational action plays a key role in selective imitation in 14-month-olds.

## Introduction

It is a demanding task for infants to filter relevant information from the enormous amount of input they receive from their environment. Nevertheless, one-year-old infants already selectively use this information to guide their own action production. A prominent example is the phenomenon of selective imitation. In a widely recognized study, Gergely, Bekkering, and Király [1] adapted a paradigm introduced by Meltzoff [2] and showed that 14-month-olds imitated an unconventional action (i.e., illuminating a lamp by touching it with one's head) if the model deliberately chose this action as a means for goal achievement (i.e., when the model's hands were free). In contrast, the likelihood of imitation was considerably reduced when external constraints in the model's situation justified her selection of the unconventional head action (i.e., when the model's hands were occupied).

The authors proposed an intriguing explanation for this phenomenon, referring to a rational action account [3]: Infants imitated selectively because they first evaluated the rationality of the model's action, taking into account her goal as well as the means available to her to achieve it under the given situational constraints, and then conducted the same means-ends analysis to guide their own action (which was always performed under unconstrained conditions). In more specific terms, this means that infants presumably inferred that the model had good reasons to freely choose to perform the unusual action in the hands-free condition. As a result, the majority of infants imitated the head touch. By contrast, in the hands-occupied condition, infants did not imitate the head touch – presumably because they understood that the model was forced to use the unusual means (head) whereas they themselves were free to use the usual means (hands).

This work is since regarded as evidence that infants draw rational inferences which influence whether they imitate a model's behavior or not (e.g. [4]–[8]). In the current study, however, we present evidence that such a complex explanation is not needed, and we propose an alternative approach to account for selective imitation in the presence of situational constraints. A closer look at the paradigm used by Gergely and colleagues [1] reveals that the hands-free and the hands-occupied condition not only differed with respect to different situational constraints, but also with respect to the saliency of context stimuli which went along with these constraints.

In both conditions, the model put on a blanket before illuminating the lamp. In the hands-free condition, she only loosely put it over her shoulders. In the hands-occupied condition, however, she pretended to be cold and wrapped herself in the blanket, holding it together from underneath in front of her torso. The blanket thus completely covered her upper body, forming an eye-catching outfit. On top of the unusual head action performed in both conditions, there was thus an additional unusual and therefore salient feature in the hands-occupied condition. This feature may, as a perceptual distractor, have competed with the head action, drawing the infant's attention away from it. Accordingly, we propose a perceptual distraction approach to account for infants' selective imitation. While the importance of directing the infant's perception towards the model's action is widely acknowledged in imitation paradigms (e.g. [2]), systematic research on the influence of perceptual processes on selective imitation is lacking.

**Figure 1:**
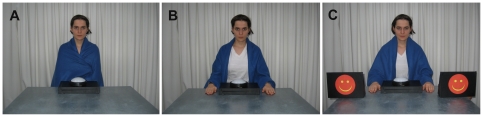
Experimental setup. The model before performing the head touch action in the hands-occupied and hands-occupied familiarization condition (A), the hands-free condition (B), and the hands-free distraction condition (C).

We tested the hypothesis of perceptual distraction from the head action due to the presence of salient context stimuli in the modeling phase by introducing two manipulations in addition to the original conditions (see Figure 1). First, in a new version of the hands-occupied condition (*hands-occupied familiarization condition*), we aimed to reduce perceptual distraction from the strange and unexpected appearance of the model by familiarizing the infants with the sight of this appearance during the 5-minute warm-up phase which in all conditions preceded the imitation task. Second, in a new version of the hands-free condition (*hands-free distraction condition*), we aimed to enhance perceptual distraction by placing red smileys on the table.

For these two additional conditions, opposing outcomes can be hypothesized from the two alternative approaches, rational action and perceptual distraction. According to the rational action approach, infants will imitate the head action in the hands-occupied familiarization condition as infrequently as in the original hands-occupied condition. They should infer that the model performed this unusual action due to situational constraints (i.e., being wrapped in the blanket). If anything, the fact that the model was already wrapped in the blanket and could not use her hands during the warm-up phase should make these situational constraints even more obvious. By contrast, according to the perceptual distraction approach one would predict the opposite – that infants will show more imitation of the head action in the hands-occupied familiarization condition than in the original hands-occupied condition. Once familiarized with the sight of the covered model, they should be in a position to focus on the unusual head action in a manner similar to in the hands-free condition.

Following the same logic, the two approaches also lead to opposite predictions in the hands-free distraction condition. Rational action predicts that infants will imitate the head action because the model's action is not constrained in any obvious way. By contrast, perceptual distraction predicts that the smileys are salient context stimuli which will distract the infants from the target action, leaving less capacity for encoding, and hence imitating it. The likelihood of imitation will thus be reduced.

## Methods

### Participants

Fifty-eight 14-month-olds (*M* = 13 months, 27 days; range 13.15 to 14.15) participated in the experiment. They were randomly assigned to one of four experimental groups (hands-occupied: *n* = 14; hands-free: *n* = 14; hands-occupied familiarization: *n* = 15; hands-free distraction: *n* = 15). Ten additional infants were tested, but had to be excluded from the final sample due to parental interference, fussiness, procedural errors, or lack of interest. The experimenter was a female adult. Infants were recruited from a database of parents who had agreed to participate in infant studies. Parents gave informed written consent prior to the experiment. The study was approved by the local ethics committee of the University of Leipzig, and conducted in accordance with the Declaration of Helsinki.

### Materials

A lamp (diameter 14 cm, height 6 cm) which was fixed on a black panel and could be illuminated by touching, and a blue blanket (145×190 cm) served as material. Additionally, in the hands-free distraction condition, two black boxes (length 25 cm, height 17.5 cm), each with a red smiley centrally attached, were used. The infants' and experimenter's behavior were recorded by two cameras.

### Procedure and Design

The experimenter entered the test room together with the infant and one parent. All conditions started with a 5-minute warm-up phase during which the experimenter played with the infant using a soft ball. The infant was then seated on the parent's lap on one side of a table, and the experimenter sat on the opposite side with the lamp in front of her. Experimenter and infant continued playing with the ball for a minute. In the following experimental phase, the experimenter called the infant by his or her name and said: ‘Look what I am doing!’ She then bent down, illuminated the lamp for 2 seconds using her forehead and returned to the upright position. This sequence was repeated three times. Subsequently, the experimenter put the lamp in front of the infant, said: ‘Now you can play with it!’ and left the room. The child was given 60 seconds to explore the lamp [9]. As in previous studies [1], [2], an action was coded as a head touch if the infant came within a minimum distance of 10 cm of the lamp with his or her head. Infants were randomly assigned to one of the following four conditions (Figure 1).

#### Hands-occupied

At the beginning of the experimental phase, the experimenter pretended to be cold and wrapped herself in a blanket. Her hands were occupied holding this blanket while she illuminated the lamp.

#### Hands-free

At the beginning of the experimental phase, the experimenter put a blanket loosely around her shoulders. Her hands were visible and free while she illuminated the lamp.

#### Hands-occupied familiarization

At the beginning of the warm-up phase, the experimenter pretended to be cold and wrapped herself in a blanket. From this moment on, her hands were occupied holding the blanket. Like in the other conditions, she played with the infant, but in this condition without using her hands (first by kicking the ball and after they sat down at the table by relying on the parent's help). In the experimental phase, she continued to hold the blanket. Her hands were thus occupied while she illuminated the lamp.

#### Hands-free distraction

At the beginning of the experimental phase, the experimenter put a blanket loosely around her shoulders. She then put two smilies to her left and right on the table. Her hands were free and visible while she illuminated the lamp.

In all conditions, the experimenter laid the blanket aside after she had performed the head action and before she put the lamp in front of the infant.

## Results

All infants touched the lamp with their hands (for similar results see [9], [16]). Critically, however, only part of the infants imitated the head touch. For the hands-occupied and the hands-free conditions, we replicated the results obtained in the original study [1]. Ten out of 14 infants (71.4%) imitated the head touch in the hands-free condition, but only 4 out of 14 (28.6%) in the hands-occupied condition, *χ^2^*(1,*N* = 28) = 5.14; *p*<.05.

**Figure 2:**
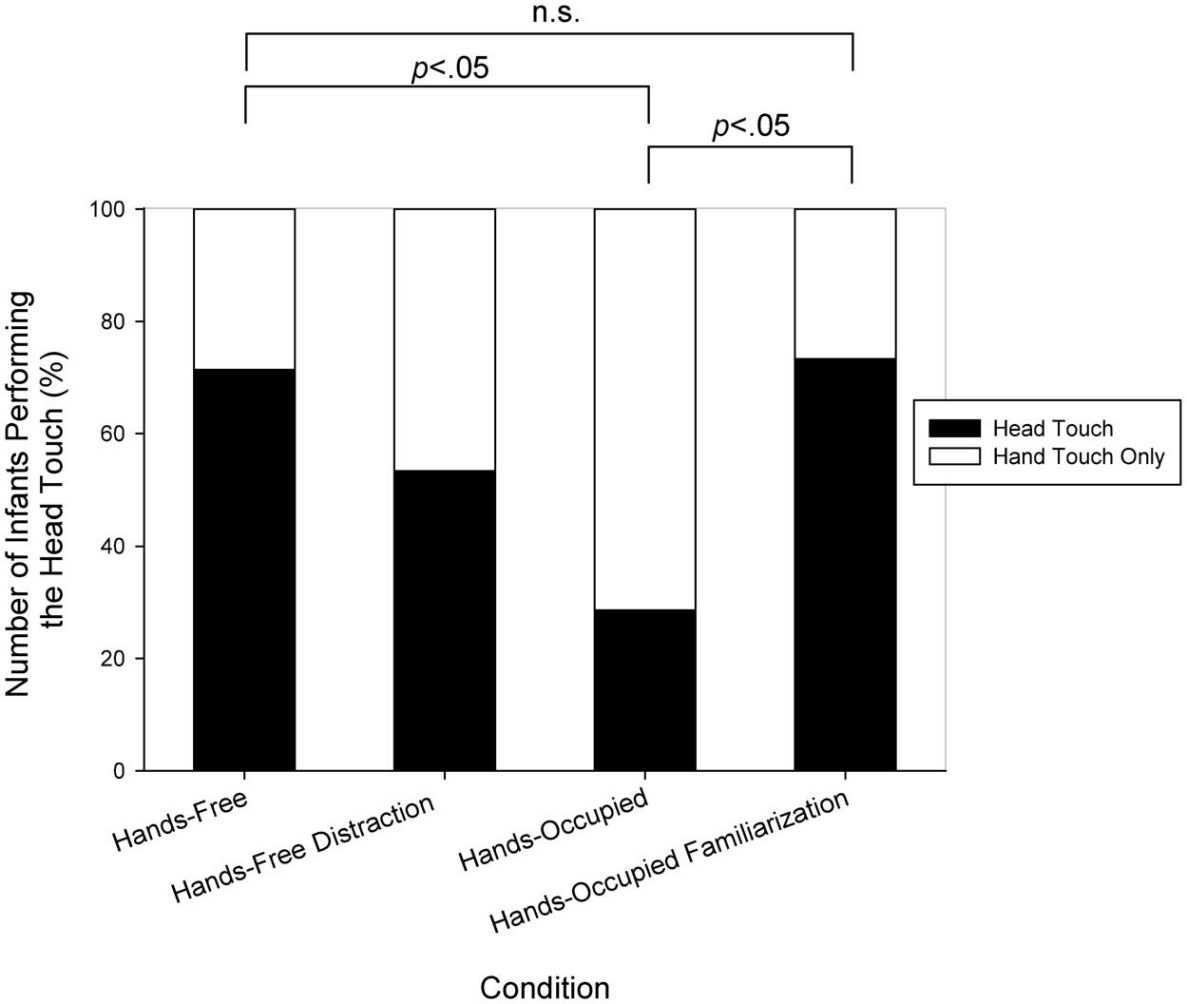
Results. Percentage of infants performing a head touch in each of the four experimental conditions. The original conditions are represented by the first (hands-free) and the third (hands-occupied) column.

More notably, the additional manipulations had significant effects as well (see Figure 2): Although the model's hands were occupied in the hands-occupied familiarization condition, 11 out of 15 infants (73.3%) imitated the head touch. The likelihood of imitation was thus significantly higher than in the original hands-occupied condition, *χ^2^*(1,*N* = 29) = 5.81; *p*<.05, and did not differ from the original hands-free condition, *χ^2^*(1,*N* = 29) = 0.01, *p* = .91.

Furthermore, although the model's hands were free in the hands-free distraction condition, only 8 out of 15 infants (53.3%) imitated the head touch. In the ranking of conditions, the likelihood of imitation thus fell between the two original conditions. The difference to the original hands-free condition, however, failed to reach significance, *χ^2^*(1,*N* = 29) = 1.8; *p* = .18). The difference to the original hands-occupied condition was not significant either, *χ^2^*(1,*N* = 29) = 1.0; *p* = .32). However, if the conditions were ranked in the following order, hands-free, hands-free distraction, hands-occupied, there was a significant correlation between the performance of the head touch (i.e., head touch or no head touch) and condition: Across the three conditions, there was a significant increase in the likelihood of imitation, *d* = .32; *p*<.05, Somer's d coefficient.

## Discussion

In previous work, selective imitation in 14-month-olds has been explained by the infants' ability to apply the principle of rational action [1]. However, the results of the present study suggest that a simpler approach, based on perceptual distraction, can explain these findings. The results show that the main factor that modulates the likelihood of infants imitating an unusual action was the presence or absence of a perceptual distractor. In the original study, such a distractor was present in form of the blanket which completely covered the model's upper body in the hands-occupied condition, but not in the hands-free condition. In the present study, the saliency of this distractor was reduced by familiarizing infants with its presence (hands-occupied familiarization condition) and the likelihood of imitation was now in the same range as under unconstrained conditions (original hands-free condition). However, when a distractor was present (original hand-occupied condition and hands-free distraction condition), the likelihood of imitation declined. Although the results in the hands-free distraction condition were less clear-cut, the overall comparison with the two original conditions and the results in general show that the likelihood of imitating the unusual action depends on the saliency of context stimuli in the modeling phase and not on the feasibility of rational accounts of the model's and the infant's own action.

Up to now, many studies have demonstrated that the manipulation of saliency affects whether and what kind of information infants perceive, and that selective perception in turn influences action performance [10]–[14]. There is a gradual increase in the amount of information that can be processed over developmental age, but in six-month-olds, local stimulus enhancement already improves performance [15]. It is thus not surprising that a saliency manipulation during the modeling phase results in selective imitation, even if the likelihood of imitation was furthermore modulated by the presence or absence of situational constraints. However, beyond demonstrating a strong role for perceptual factors, our findings even seem to rule out a rational imitation approach. As outlined in the introductory section, rational imitation predicts the opposite of what we observed: In the hands-occupied familiarization condition, infants experienced the model's external constraints much more clearly and explicitly than in the original hands-occupied condition since, in the warm-up phase, the model already did not use her hands to play after she had wrapped herself in the blanket. The model's situational constraints were thus manifest for a longer period of time and during a phase of direct interaction with the infant. Rational imitation must therefore predict at least a similarly low, or an even lower likelihood of imitation as observed in the original hands-occupied condition. The present results indicate the opposite, however: The likelihood of imitation was high, as predicted by perceptual distraction.

Another alternative explanation for selective imitation in infants has recently been proposed by Paulus, Hunnius, Vissers, and Bekkering [16]. They refer to the approach of motor resonance [17] and postulate that a match between the model's and the infants' body posture is critical to activate the motor program of the modeled action: When infants perform a head touch, they always have their hands on the table to maintain a stable position. In the study by Gergely et al. [1], the model took this position only in the hands-free condition. Only in this condition there was thus a match between body postures, and consequently, the likelihood of imitation was high. The approach of motor resonance cannot, however, explain imitation in our hands-occupied familiarization condition because in this condition, there was no match between the model's and the infants' body posture. Conversely, the perceptual distraction approach is in accordance with the results Paulus et al. [16] report for their test conditions: The likelihood of imitation was low when the model held her hands up in the air while performing the head touch (the infants were presumably distracted by this unusual behavior) and when she was totally wrapped in a blanket held by a button (strange appearance). The likelihood of imitation was high when the model first played with two soft balls and then kept one ball in each hand while performing the head touch (infants could focus their attention on the target action when the model stopped playing with the balls).

With some adaptations, the paradigm used by Gergely et al. [1] has also been applied to demonstrate selective imitation in 12-month-old infants [9], enculturated chimpanzees [18], and even in domesticated dogs [19]. Whereas the authors of these studies follow the theoretical framing of the original study and refer to the rational action approach to explain their results, the selection of participants suggests that a less-demanding perceptual interpretation might be more appropriate for these studies as well. Such an alternative interpretation of rational imitation in domesticated dogs has already been reported by Kaminski et al. [20]. In light of the tendency to ascribe other sophisticated cognitive abilities to infants of very young age (e.g., false belief understanding to one-year-olds, see [21] for a review), we vote to acknowledge the potential contribution of perceptual processes in tasks that are designed to test higher cognitive abilities (see [22] for a similar view).

To sum up, the present study demonstrates that the phenomenon of selective imitation in 14-month-olds does not require the demanding rational action approach, but can be comprehensively explained by a perceptual distraction approach. More importantly, our findings actually rule out the possibility that the rational action approach can serve as an explanation for the observed effects. In contrast to that approach, infants nonselectively imitate new and unusual means for goal achievement – provided that perceptual distraction by context stimuli present in the modeling phase is controlled in a way that allows them to encode these means. The rational action approach should thus be rethought in debates on infant imitation.
